# Ellipsoid Zone Defects in Retinal Vein Occlusion Correlates With Visual Acuity Prognosis: SCORE2 Report 14

**DOI:** 10.1167/tvst.10.3.31

**Published:** 2021-03-26

**Authors:** Tyler Etheridge, Ellen T. A. Dobson, Marcel Wiedenmann, Neal Oden, Paul VanVeldhuisen, Ingrid U. Scott, Michael S. Ip, Kevin W. Eliceiri, Barbara A. Blodi, Amitha Domalpally

**Affiliations:** 1 Department of Ophthalmology and Visual Sciences, University of Wisconsin School of Medicine and Public Health, Madison, WI, USA; 2 Laboratory for Optical and Computational Instrumentation, Center for Quantitative Cell Imaging, University of Wisconsin-Madison, Madison, WI, USA; 3 KNIME GmbH, Konstanz, Germany; 4 The Emmes Company, LLC, Rockville, MD, USA; 5 Departments of Ophthalmology and Public Health Sciences, Penn State College of Medicine, Hershey, PA, USA; 6 Doheny Eye Institute, University of California Los Angeles Stein Eye Institute, Los Angeles, CA, USA; 7 McPherson Eye Research Institute, University of Wisconsin-Madison, Madison, WI, USA; 8 Department of Medical Physics, University of Wisconsin-Madison, Madison, WI, USA

**Keywords:** anti-VEGF, semiautomated, machine learning, ellipsoid zone, macular edema, optical coherence tomography, retinal vein occlusion

## Abstract

**Purpose:**

To evaluate the association between ellipsoid zone (EZ) on spectral domain optical coherence tomography (SD-OCT) and visual acuity letter score (VALS) in participants with retinal vein occlusion in the Study of Comparative Treatments for Retinal Vein Occlusion 2.

**Methods:**

SD-OCT scans of 362 participants were qualitatively assessed at baseline and months 1, 6, 12, and 24 for EZ status as normal, patchy, or absent. The thickness of EZ layer in the central subfield was also obtained using machine learning.

**Results:**

EZ assessments were not possible at baseline due to signal blockage in >75% of eyes. At month 1, EZ was normal in 37.6%, patchy in 48.1%, and absent in 14.3%. EZ was measurable in 48.7% with a mean area of 0.07 ± 0.16 mm^2^. Mean VALS was better in eyes without an EZ defect compared to eyes with an EZ defect (*P* < 0.0001 at all visits). EZ defect at month 1 was associated with poorer VALS at all follow-up visits (*P* < 0.0001).

**Conclusions:**

Both qualitative and quantitative assessments of EZ status strongly correlated with VALS. Absence of EZ was associated with poorer VALS at both corresponding and future visits, with larger areas of EZ loss associated with worse VALS.

**Translational Relevance:**

Assessment of EZ can be used to identify patients with potentially poor response in eyes with retinal vein occlusion.

## Introduction

Retinal vein occlusion (RVO) is the second most common form of retinal vascular disease, with an estimated prevalence between 0.4% and 1.6% worldwide.^1^^,^^2^ Occlusion may occur in the central retinal vein or one of its branches with subsequent upregulation of vascular endothelial growth factor (VEGF), increased vessel permeability, loss of the blood-retinal barrier, and accumulation of fluid in the retinal and subretinal layers. Macular edema is the most common vision-threatening complication, with a prevalence of up to 15% of eyes with branch RVO (BRVO) and 30% with central RVO (CRVO) and hemi-retinal vein occlusion (HRVO).^3^^,^^4^ In eyes with macular edema due to RVO, changes in the photoreceptor layer can be identified as a disruption or defect in the ellipsoid zone (EZ), as seen on spectral domain optical coherence tomography (SD-OCT). An EZ defect can occur in treatment-naive eyes with macular edema and in anti-VEGF–treated eyes with persistent or no macular edema.^5^ The EZ, previously named the photoreceptor inner segment–outer segment junction, is visualized as a hyperreflective band in the outer retina on SD-OCT.^6^ The EZ is thought to be formed by mitochondria within the ellipsoid layer of the outer portion of the inner segments of the photoreceptors; thus, the EZ is an essential part of the visual system, well suited for a structural OCT surrogate parameter.^7^ The integrity of the EZ has been associated with visual acuity (VA) and visual outcomes in RVO,^5^^,^^8^ likely due to photoreceptor integrity and function.^9^

Intravitreal anti-VEGF therapy has shown to be safe and effective for the treatment of macular edema secondary to RVO.^10^^,^^11^ The anti-VEGF medications aflibercept and bevacizumab are commonly used for the treatment of macular edema secondary to CRVO.^12^^,^^13^ The Study of Comparative Treatments for Retinal Vein Occlusion 2 (SCORE2) recently showed that, with regard to visual acuity letter score (VALS), intravitreal bevacizumab was noninferior to aflibercept in patients with CRVO and HRVO.^14^ Although studies have demonstrated that visualization of EZ integrity improves in eyes with macular edema secondary to RVO receiving anti-VEGF therapy, and EZ integrity may predict visual outcomes,^15^^,^^16^ there is a paucity of large clinical trial data with long-term follow-up. In addition, assessment of EZ is mostly based on integrity of EZ and not a quantitative assessment of area or thickness. Accurate surrogate parameters improve the selection of eyes for clinical trials, establish therapeutic outcomes, and influence clinical management. Therefore, we sought to evaluate the EZ integrity assessed by SD-OCT–generated en face thickness maps and its association with VALS in eyes with macular edema secondary to CRVO or HRVO in the SCORE2 trial using a semiautomated, machine learning approach.

## Methods

### Study Participants

Study data were obtained from SCORE2, a multicenter, prospective, randomized noninferiority trial of eyes with macular edema secondary to CRVO or HRVO comparing intravitreal bevacizumab versus aflibercept (clinicaltrials.gov identifier NCT01969708).^14^ The study was approved by the institutional review boards associated with each center and adhered to the tenets of the Declaration of Helsinki. All participants provided written informed consent. The SCORE2 design and methods have been previously described.^17^ In summary, 362 participants were randomized to receive either intravitreal bevacizumab or aflibercept. The study visits were conducted with treatment provided per protocol from baseline through month 12 and then at the discretion of the investigator thereafter. Inclusion criteria were center-involved macular edema, defined as central subfield thickness (CSF) of ≥300 µm (or ≥320 µm if measured on a Heidelberg Spectralis Machine; Spectralis Heidelberg Engineering, Heidelberg, Germany), and VALS ≥19 Early Treatment Diabetic Retinopathy Study (ETDRS) letters (approximately 20/400) and ≤73 letters (approximately 20/40). Additionally, media clarity was required for imagine acquisition, and eyes with cataract were excluded per study criteria.

### Data Collection

All SD-OCT scans were acquired by certified photographers using the SCORE2 reading center (Fundus Photograph Reading Center, University of Wisconsin-Madison) approved protocol with either a Carl Zeiss Meditec Cirrus (Carl Zeiss Meditec, Dublin, CA, USA) or a Heidelberg Spectralis (Spectralis Heidelberg Engineering, Heidelberg, Germany) OCT machine on dilated eyes.^17^ The Zeiss macular volume scans were 6 mm and comprised 512 A-scans and 128 B-scans, and the Heidelberg scans were 20 × 20 degrees and comprised 512 A-scans and 97 B-scans. Imaging was obtained from one eye of each study participant. SD-OCT scans were evaluated at baseline, month 1 (M01), month 6 (M06), month 12 (M12), and month 24 (M24) for all participants. Demographic data were obtained from eligible study participants through review of medical records. Best-corrected VALS using the ETDRS protocol was recorded at all study visits.

### Ellipsoid Zone Analysis

**Figure 1.**
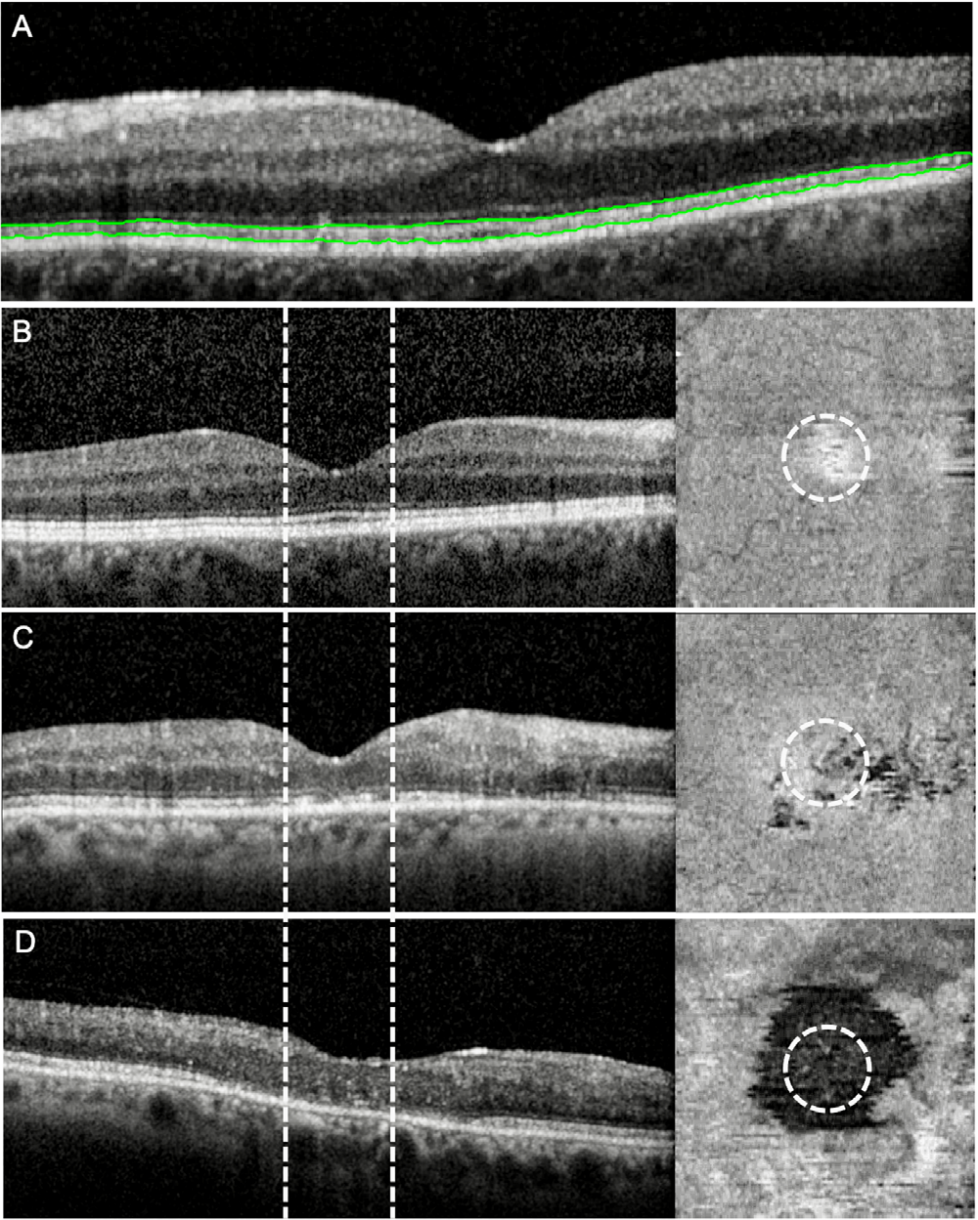
Representative SD-OCT scans. (A) SD-OCT scan with segmented EZ (*green lines*). SD-OCT scans with corresponding en face thickness maps demonstrating normal (B), patchy (C), and absent (D) EZ within the central subfield (*dashed line and circle*).

The methods to evaluate the EZ as assessed by SD-OCT have been previously described in detail.^18^ To summarize, all scans were converted to DICOM format for segmentation within a custom-developed MATLAB (MathWorks, Natick, MA, USA) segmentation platform (Fig. 1A).^19^ The inner border of the second outer hyperreflective band (EZ layer) and the inner border of the third outer hyperreflective band (retinal pigment epithelium [ RPE]) were selected as the EZ layer boundaries for segmentation.^6^ The EZ layer was segmented in the CSF, and en face thickness maps were generated within a customized workflow (Figs. 1B–D). Image quality was assessed for clear identification of outer retinal layers. Distinction of visible EZ and EZ defects was required for completion of segmentation.

Regions of EZ defect within the CSF were identified from en face thickness maps via a machine learning–generated classifier. The Fiji plugin,^20^ Trainable Weka Segmentation,^21^ was used to generate the classifier applying binary pixel classification. Areas of EZ defect appear as dark areas on the thickness maps compared to bright areas with normal EZ. The machine learning classifier was applied via a customized workflow using the open-source data analytics platform Konstanz Information Miner (KNIME), version 3.7.2 (Zurich, Switzerland).^22^ Our previous study demonstrated excellent agreement and reliability between semiautomated EZ defect area measurements and manual measurements performed by certified graders, with an intraclass correlation coefficient of 0.90 and average bias of 0.01 mm^2^ (95% confidence interval [CI], 0.17–0.31).^18^ The minimum area of EZ defect was defined as 0.004 mm^2^ based on the lowest limit of area measurability used within reading center grading protocols (e.g., drusen circle C_0_ established by the Age-Related Eye Disease Study Research Group). Values less than 0.004 mm^2^ were considered zero. The maximum area of EZ defect was 0.78 mm^2^ based on the area of the CSF.

Qualitative grading of the EZ status was performed as well. The status of the EZ within the CSF on SD-OCT was graded per SCORE2 protocol via certified graders at the central reading center (Fundus Photograph Reading Center, University of Wisconsin-Madison). The appearance of the EZ within the CSF was graded using a three-step scale as normal (Fig. 1B), patchy (Fig. 1C), absent (Fig. 1D), or cannot grade. The complete methods of this three-step scale have been described in detail in a pilot study of 42 randomly selected participants in the predecessor SCORE trial.^18^ Intergrader agreement on the presence versus absence of EZ defect was 79%, with a κ of 0.58 (95% CI, 0.44–0.72)

### Statistical Analysis

Categorical variables were summarized as percentages and continuous variables were summarized as means. The mean values for EZ area presented at each visit are based on one measurement per study participant per visit and do not include multiple measurements per participant at a visit. Unpaired two- tailed *t*-tests were calculated to compare mean VALS grouped by the presence or absence of an EZ defect. We calculated a longitudinal mixed model with measurements in at least one of all four follow-up visits that regressed VALS change from baseline on contemporaneous continuous EZ defect area categorical visit number (M01, M06, M12, M24) and their interaction, with an autoregressive correlation structure for each participant. A second longitudinal mixed model regressed VALS change from baseline on categorical contemporaneous EZ in the CSF as assessed by graders (absent, patchy, and normal EZ), categorical visit (M01, M06, M12, M24), and their interaction, with an autoregressive structure for each study participant.

In addition, we investigated the association between machine learning–generated EZ defect area measurements and qualitative EZ grading. All qualitative grading was expressed as categorical variables, and EZ defect area was expressed as a continuous variable. Associations between qualitative and quantitative assessments were calculated using one-way analysis of variance. After Bonferroni correction for multiple comparisons, a *P* value of less than 0.002 was considered significant for all statistical tests. Statistical analysis was performed using SAS software version 9.4 (SAS Institute, Cary, NC, USA).

## Results

### SD-OCT Scans Analyzed

**Figure 2.**
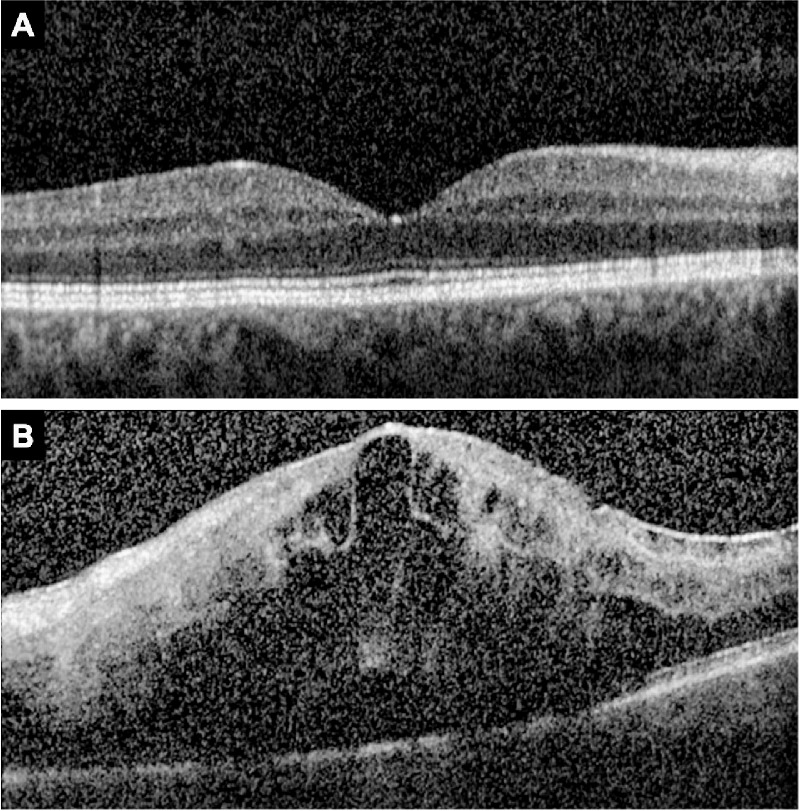
Representative SD-OCT scans. (A) SD-OCT scan with gradable EZ. (B) SD-OCT scan with ungradable EZ due to signal blockage by hemorrhage and fluid. Note drop out of Bruch's membrane due to poor signal strength.

All 362 SCORE2 study eyes were included in this study. Assessment of the EZ was not possible at baseline due to a >75% rate of ungradable SD-OCT volume scans. The number of ungradable scans was 121 of 353 (34.3%) at M01, 36 of 354 (10.2%) at M06, 28 of 327 (8.6%) at M12, and 21 of 226 (9.3%) at M24. On qualitative grading, SD-OCT scans were deemed ungradable due to signal blockage by hemorrhage or fluid resulting in poor signal intensity in the outer retinal layers (Fig. 2).

### Presence or Absence of EZ Defect and VALS

Before generating an area output, the algorithm assessed presence or absence of EZ defect as a binary variable (defined as an area >0.004 mm^2^). Based on this definition, EZ defect was absent in 48.7% (113/232) at M01. The percentage of eyes with a defect decreased to 39.0% (120/308) at M06 and 32.8% (98/299) at M12. At M24, the percentage of eyes with an EZ defect increased to 44.4% (91/205). An EZ defect at M01 was negatively correlated with VALS at subsequent visits: M06 (*r* = −0.35, *P* < 0.0001), M12 (*r* = −0.34, *P* < 0.0001), and M24 (*r* = −0.39, *P* < 0.0001).

### EZ Defect Area and VALS

**Figure 3.**
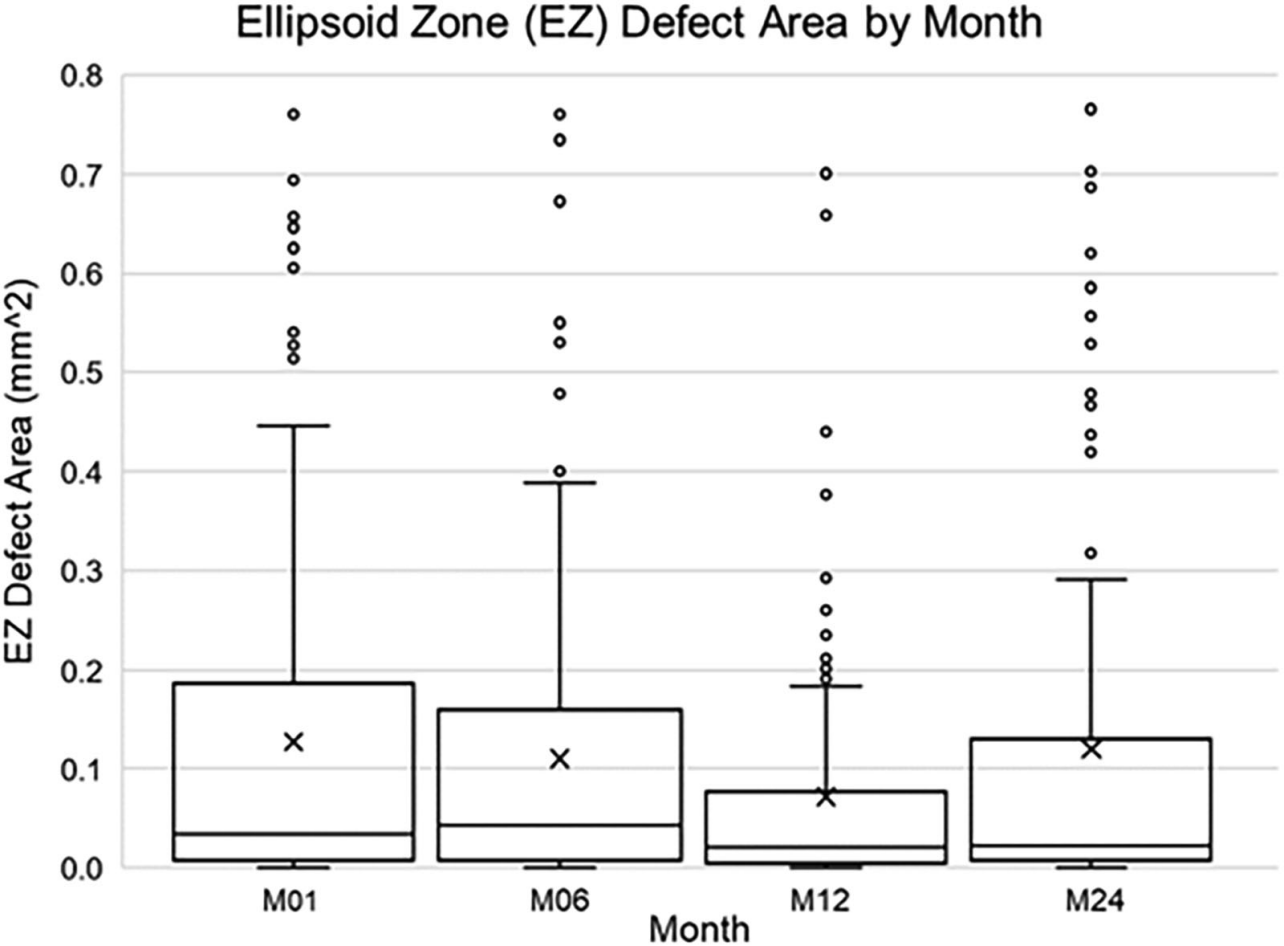
EZ defect area (mm^2^) by month. Mean ± SD EZ defect area was 0.07 ± 0.16 mm^2^ at M01, 0.05 ± 0.12 mm^2^ at M06, 0.03 ± 0.09 mm^2^ at M12, and 0.06 ± 0.16 mm^2^ at M24.

The mean ± SD area of EZ defect within the CSF was 0.07 ± 0.16 mm^2^ at M01 (Fig. 3). At M06 and M12, the mean area of EZ defect decreased to 0.05 ± 0.12 mm^2^ and 0.03 ± 0.09 mm^2^. The mean area increased to 0.06 ± 0.16 mm^2^ at M24. There was no association between the number of injections received or the randomly assigned anti-VEGF medication and EZ defect area. The sample size differs among the different visits due to inability to measure EZ defect and attrition of study population. However, a subset of 122 patients with nonmissing EZ defect values for all four follow-up visits did not demonstrate striking differences in mean ± SD EZ defect area (M01, 0.07 ± 0.14 mm^2^; M06, 0.04 ± 0.11 mm^2^; M12, 0.03 ± 0.10 mm^2^; and M24, 0.06 ± 0.15 mm^2^).

**Figure 4.**
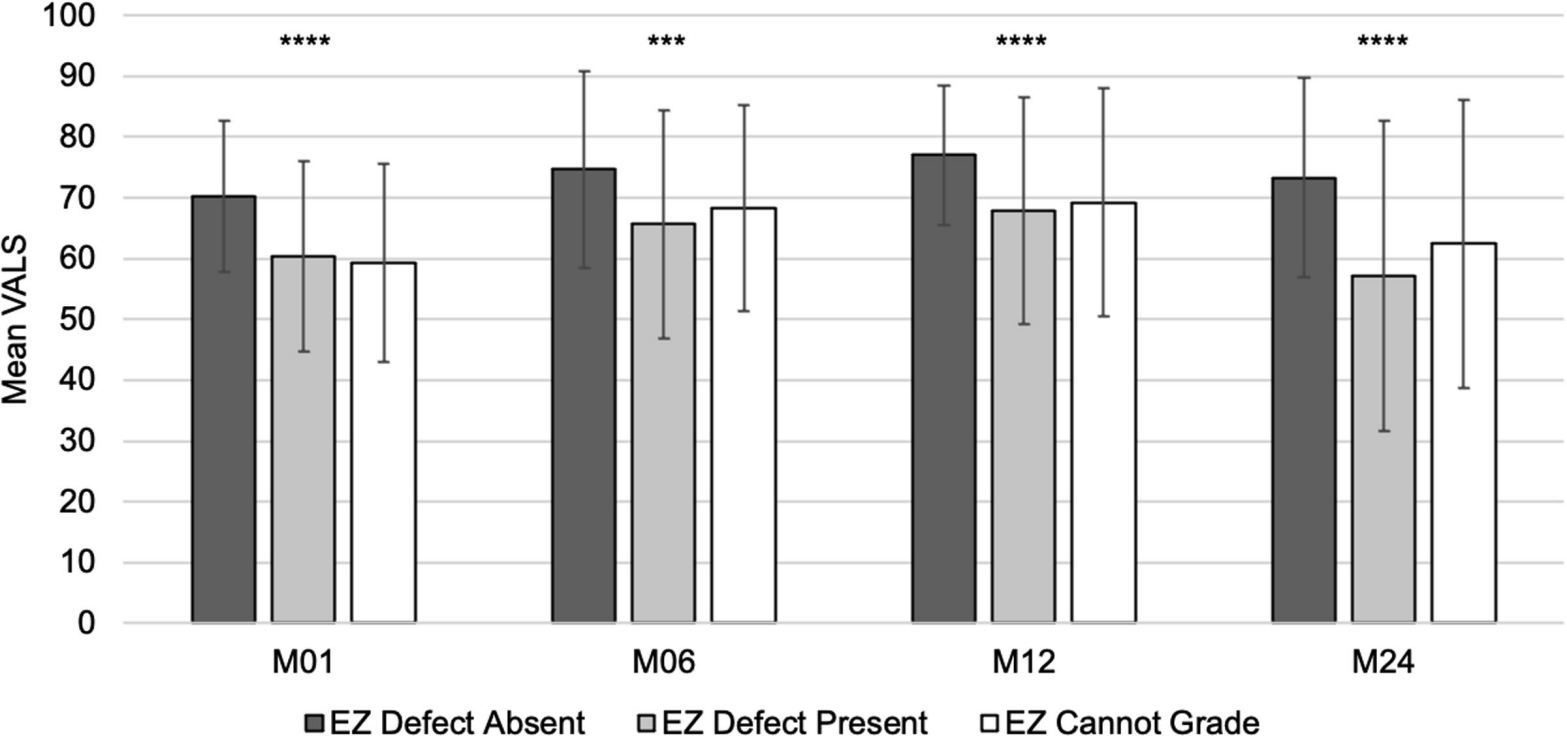
VALS by month and the presence or absence of an EZ defect. At all subsequent visits, mean ± SD VALS was significantly different in eyes without an EZ defect at M01 compared to eyes with an EZ defect and also eyes with ungradable EZ defect.

The mean ± SD VALS was 63.3 ± 15.7 at M01. VALS improved to 69.3 ± 17.6 at M06 and 71.1 ±1 7.2 at M12. At M24, mean VALS worsened to 64.3 ± 23.0. The mean ± SD VALS at all subsequent visits was greater in eyes without an EZ defect at M01 compared to those with an EZ defect and also those that could not be graded for an EZ defect: M01 (70.2 ± 12.5 vs. 60.4 ± 15.7 and 59.3 ± 16.3, *P* < 0.0001), M06 (74.6 ± 16.1 vs. 65.6 ± 18.7 and 68.3 ± 17.01, *P* = 0.0001), M12 (77.0 ± 11.5 vs. 67.8 ± 18.7 and 69.2 ± 18.7, *P* < 0.0001), and M24 (73.3 ± 16.4 vs. 57.2 ± 25.5 and 62.4 ± 23.6, *P* < 0.0001) (Fig. 4). At each subsequent visit, eyes without an EZ defect at M01 had better vision than eyes that either had an EZ defect or were ungradable for an EZ defect. The association between EZ defect area and contemporaneous VALS was significant at every visit number and strengthened over time. More specifically, an increase of 0.05 mm^2^ in EZ defect area was associated with a 0.61 lowering in mean VALS change from baseline at M01 (*P* = 0.0100), 0.99 at M06 (*P* < 0.001), 1.07 at M12 (*P* = 0.0007), and 2.17 at M24 (*P* < 0.001). There was no association between the number of injections received or the anti-VEGF medication provided and EZ defect area.

### EZ Defect Area and CSF Thickness

The mean ± SD CSF thickness was 299.7 ± 118.5 µm at M01. CSF thickness improved to 260.5 ± 103.6 µm and 253.9 ± 114.1 µm at M06 and M12, then worsened to 310.7 ± 160.6 µm at M24. The mean CSF thickness and EZ defect area were not correlated at M01, M06, and M12 (all *P* > 0.002). At M24, EZ defect area positively correlated with CSF thickness (*r* = 0.39, *P* < 0.0001).

### Qualitative EZ Grade

Qualitative assessment of the EZ in the CSF as normal, patchy, or absent on SD-OCT showed 37.6% (87/231) of scans were normal, 48.1% (111/231) were patchy, and 14.3% (33/231) were absent at M01 (Table). The percentage of scans that were graded as normal, patchy, or absent at M06 was (40.8%, 48.9%, and 10.3%), M12 (39.6%, 45.3%, and 15.1%), and M24 (54.0%, 35.5%, and 10.5%), respectively.

**Table.** EZ Defect Area by Qualitative EZ Status Grade by Month

		EZ Defect Area			
Month	EZ Status	*N*	Mean	SD	*P* Value
M01	Normal	87	0.01	0.03	<0.0001
	Patchy	111	0.07	0.14	
	Absent	33	0.24	0.26	
M06	Normal	126	0.005	0.04	<0.0001
	Patchy	151	0.05	0.09	
	Absent	32	0.21	0.24	
M12	Normal	118	0.0003	0.001	<0.0001
	Patchy	135	0.03	0.06	
	Absent	45	0.12	0.17	
M24	Normal	108	0.03	0.11	<0.0001
	Patchy	71	0.07	0.16	
	Absent	21	0.23	0.26	

### Qualitative EZ Grade and EZ Defect Area

The qualitative grade was entirely done by human graders independent of the algorithm-generated EZ defect area. There was a larger EZ defect area at all time points for the grade of absent and patchy EZ compared to normal (*P* < 0.0001 for all time points) (Table). At M01, the mean ± SD EZ defect area was 0.24 ± 0.26 mm^2^ for the grade of absent EZ (*n* = 33), 0.07 ± 0.14 mm^2^ for patchy (*n* = 111), and 0.01 ± 0.03 mm^2^ for normal (*n* = 87, *P* < 0.0001). Twenty-two eyes had a measurable EZ defect area but received a qualitative grade of normal EZ at M01. The mean CSF thickness was not associated with qualitative EZ status grades at M01, M06, and M12.

### Qualitative EZ Grade and VALS

The association between VALS change from baseline as a function of study visit (M01, M06, M12, M24) and qualitative EZ grade (absent, patchy, normal) was significant at all study visits (*P* < 0.0001) in the longitudinal mixed model. Specifically, mean VALS change from baseline increased from the grade of absent to patchy to normal EZ at M01 (12.5, 13.3, 15.4 letters), M06 (13.7, 19.5, 20.8 letters), M12 (19.4, 21.1, 22.5 letters), and M24 (10.0, 11.0, 19.8 letters).

## Discussion

Macular edema is the most common vision-threatening complication of RVO.^3^^,^^4^ In eyes with macular edema due to RVO, disruption of the photoreceptor integrity, which is an essential part of the visual pathway, can be visualized as a disintegration of the EZ on SD-OCT.^9^ The integrity of the EZ has therefore been associated with VA outcomes in RVO.^5^^,^^8^ In the current study, the presence of an EZ defect was significantly correlated with poor VALS at both the corresponding visit and subsequent visits. In addition, there was a significant association between area of EZ loss and VALS at every visit. Further, we utilized a three-step clinical scale for EZ defect that has a comparable correlation to VALS change. While the semiautomated measurements of EZ loss are more useful for clinical research, the qualitative approach using the three-step scale will help clinicians with therapeutic management. With a semiautomated assessment of EZ defect, such as our machine learning workflow, future applications of EZ assessment may be able to assist in the selection of eyes for clinical trials and serve as a surrogate endpoint in clinical trials.

Our results showed that the presence of an EZ defect at M01 predicted worse VALS at subsequent visits. Inability to grade EZ at M01 was also associated with worse VALS at subsequent visits. A retrospective cohort study by Chan et al.^23^ examining multiple SD-OCT parameters in 84 patients with macular edema secondary to CRVO receiving anti-VEGF therapy demonstrated that reduced visualization of the EZ over 3 months predicted worsening of VA through 12 months. Similarly, Tang et al.^24^ analyzed 63 eyes with macular edema secondary to BRVO or CRVO receiving anti-VEGF therapy in a retrospective cohort study and found that EZ disruption at baseline and change in EZ disruption at 1 month were predictive of VA and likelihood of VA improvement or decline at 3 months. Finally, the retrospective cohort study by Fujihara-Mino et al.^25^ of 57 eyes with macular edema secondary to BRVO or CRVO receiving anti-VEGF therapy showed that intact EZ at the time of resolution of macular edema correlated with improved final VA at a mean follow-up time of 17.8 months. Taken together, these data suggest that early recovery of the EZ may be a key driver of visual outcomes in RVO. Early improvement of EZ integrity visualized on SD-OCT likely represents resolution of macular edema causing disruption of the photoreceptors leading to reapproximation and/or organization of this visually essential retinal layer; however, histologic correlates are lacking and represent an area of future research.

We applied a semiautomated, machine learning workflow that previously demonstrated excellent agreement and reliability with manual measurements of EZ defect.^18^ In the SCORE2 study, a larger EZ defect area was strongly associated with the grader's qualitative assessment of absent and patchy EZ status at all time points (*P* < 0.0001). In a semiautomated analysis of the EZ in 112 eyes with macular edema secondary to BRVO or CRVO, baseline VA was inversely associated with total EZ defect and other EZ parameters, such as EZ-RPE attenuation.^26^ EZ parameters did not correlate with CSF thickness, cube volume, and average cube thickness, which are common SD-OCT parameters used in RVO clinical trials in this study. Similarly, we did not observe consistent correlations between CSF thickness and EZ defect area or qualitative EZ status grades. Although visualization of EZ integrity was assessed within the CSF and globally in the previously mentioned study, data suggest that EZ disruption within the CSF is more visual significant than elsewhere in the retina.^27^ In addition, our approach of evaluating the CSF is consistent with those employed in other studies.^24^^,^^28^

Other studies have examined the association of EZ defect with final VA after subgrouping patients according to initial VA. In a retrospective cohort study of 22 eyes subgrouped according to initial VA of either ≤20/200 or >20/200 with macular edema secondary to CRVO, the group with poorer VA had higher rates of EZ defect compared to the better VA group.^29^ Disruption of the EZ correlated with worse final VA after a mean follow-up time of 14.6 months. In a post hoc analysis of a 7-month phase 4 RVO trial, the percent disruption from the external limiting membrane (ELM) was independently associated with visual acuity at baseline. At month 7, none of the OCT surrogate parameters were independently associated with improvement in VALS.^30^ The trial had a relatively short duration compared to SCORE2 and also used a single high-resolution scan through the foveal center. The data from SCORE2 trial represent one of the largest long-term prospective clinical trial results studying the association of EZ and visual acuity and therefore provides conclusive evidence that EZ defect in retinal vein occlusion correlates with worse visual acuity.

There are limitations in the assessment of EZ layer from OCT images. Evaluation of the EZ is impaired by the presence of hemorrhage and fluid, which reduces outer retinal signal intensity.^31^ Reduced outer retinal signal intensity may produce shadowing of the outer retinal layers, creating artifact, which affects the accuracy of EZ assessment. The integrity of the EZ represents a good imaging target, since it is usually hyperreflective compared to the other zones and may be easier to assess, as shown in this study. At baseline, over 75% of SCORE2 scans were ungradable due to the presence of hemorrhage and fluid using both qualitative and quantitative approaches (Fig. 2). In Figure 2B, presence of fluid reduces the signal strength and visibility of all outer retinal layers, including the EZ. However, at M01, 65.7% of study eyes were gradable after a single anti-VEGF injection. The number of gradable scans continued to increase at M06 (89.8%) and M12 (91.4%) with protocol-defined therapy. At M24, after SCORE2 participants were treated off protocol for 1 year, the number of gradable scans decreased (90.7%). Inability to measure EZ defect, as well as attrition of the study population, resulted in sample size differences among the different study visits. However, EZ defect means and standard deviations of participants with EZ defect measurements at all four follow-up visits are consistent with those of the entire population, including those with intermittently missing data (not shown). In addition, the current semiautomated, machine learning–based workflow calculated the mean area of EZ defect but did not provide the location, orientation, and shape of the defect.

Despite these limitations, our study was performed using SD-OCT scans obtained from a large (*n* = 362) prospective, multicenter, randomized controlled trial. In the only other prospective study, Shiono et al.^15^ investigated 27 eyes with macular edema secondary to BRVO receiving anti-VEGF therapy and they showed that an EZ defect at the time of macular edema resolution correlated with lower VA at 12 months compared to eyes with no EZ defect. In addition, our study was conducted through 24 months of follow-up. The study with the longest follow-up to date had an average follow-up time of 47.4 months.^16^ However, this study only included 54 patients with treated BRVO or CRVO. EZ disruption, along with other predictable factors such as age, duration of RVO, and ischemia, was predictive of final visual outcome. Although some studies have examined the impact of final EZ integrity on final VA,^32^ we examined initial EZ integrity as a predictor of final VA based on the importance of SD-OCT–derived surrogate parameters in this population.

In conclusion, improvement in mean VALS at M01, M06, and M12 correlated with a smaller area of EZ defect. At the M24 visit, after participants had been treated off protocol for 12 months, the EZ defect area and VALS worsened. At all visits, VALS was better in eyes without an EZ defect compared to those with an EZ defect. In addition, an EZ defect at M01 predicted poorer VALS at subsequent visits. In an unprecedented number of eyes with RVO, our study demonstrates that the integrity of the EZ as visualized on SD-OCT is a promising marker for VA prognosis. Therefore, EZ integrity can serve as an important element in assessing the effectiveness of therapeutic interventions and improving disease monitoring.
